# Pressure-Dependent Structure of Methanol–Water Mixtures up to 1.2 GPa: Neutron Diffraction Experiments and Molecular Dynamics Simulations

**DOI:** 10.3390/molecules26051218

**Published:** 2021-02-25

**Authors:** László Temleitner, Takanori Hattori, Jun Abe, Yoichi Nakajima, László Pusztai

**Affiliations:** 1 Wigner Research Centre for Physics, Konkoly Thege út 29-33, H-1121 Budapest, Hungary; Temleitner.Laszlo@wigner.hu; 2 J-PARC Center, Japan Atomic Energy Agency, 2-4 Shirakata, Tokai-mura, Naka-gun, Ibaraki 319-1195, Japan; hattori.takanori@jaea.go.jp; 3 Neutron Science and Technology Center CROSS, 162-1, Shirakata, Tokai-mura, Naka-gun, Ibaraki 319-1106, Japan; j_abe@cross.jp; 4 Department of Physics, Kumamoto University, Kurokami 2-39-1, Kumamoto 860-8555, Japan; yoichi@kumamoto-u.ac.jp; 5 International Research Organization for Advanced Science and Technology (IROAST), Kumamoto University, 2-39-1 Kurokami, Chuo-ku, Kumamoto 860-8555, Japan

**Keywords:** alcohol–water mixtures, high pressure, neutron diffraction, molecular dynamics simulations

## Abstract

Total scattering structure factors of per-deuterated methanol and heavy water, CD_3_OD and D_2_O, have been determined across the entire composition range as a function of pressure up to 1.2 GPa, by neutron diffraction. The largest variations due to increasing pressure were observed below a scattering variable value of 5 Å^−1^, mostly as shifts in terms of the positions of the first and second maxima. Molecular dynamics computer simulations, using combinations of all-atom potentials for methanol and various water force fields, were conducted at the experimental pressures with the aim of interpreting neutron diffraction results. The peak-position shifts mentioned above could be qualitatively reproduced by simulations, although in terms of peak intensities, the accord between neutron diffraction and molecular dynamics was much less satisfactory. However, bearing in mind that increasing pressure must have a profound effect on repulsive forces between neighboring molecules, the agreement between experiment and computer simulation can certainly be termed as satisfactory. In order to reveal the influence of changing pressure on local intermolecular structure in these “simplest of complex” hydrogen-bonded liquid mixtures, simulated structures were analyzed in terms of hydrogen bond-related partial radial distribution functions and size distributions of hydrogen-bonded cyclic entities. Distinct differences between pressure-dependent structures of water-rich and methanol-rich composition regions were revealed.

## 1. Introduction

Hydrogen-bonded liquids are indispensable parts of our lives: one can just think of liquid water and all the aqueous solutions that surround (seawater, beverages, etc.) and compose (body fluids, biomolecules) us. Furthermore, it has to be stressed that the stability of hydrogen-bonded structures against thermodynamic variables (temperature, pressure) is a key issue from the point of view of life sciences: all living organisms are made of H-bonded constructions with an incredibly delicate balance between stability and flexibility. This is why any advance towards a better understanding of the response of H-bonded networks to temperature and pressure would be significant. Here, we intend to make a ground-breaking step in understanding the effects of GPa pressures on the structure of alcohol–water liquid mixtures by reporting the first true high-pressure diffraction experiments over the entire composition range. High-pressure diffraction experiments (i) can probe the validity of short-range interatomic potentials, and (ii) they provide information on the stability of the hydrogen-bonded network.

Methanol–water liquid mixtures at room temperature and atmospheric pressure are among the most extensively studied hydrogen-bonded liquids: recent experiments (e.g., [1]) and computer simulations (e.g., [2,3]) over the full concentration range are abound. Although quite a few studies have already appeared that considered the effect of temperature (e.g., [4,5,6]) on the structure of methanol–water liquid mixtures and the effect of pressure (e.g., [7,8,9,10]) on the structure of individual hydrogen-bonded liquids, a systematic study on the effect of high pressures is still missing. On the basis of the limited experimental (diffraction) data available, it can be suspected that lowering the temperature leads to enhanced ordering with an increasing number of hydrogen bonds [5,6], whereas increasing pressure seems to “crush” the H-bonded network of water [9,10]. As a result of increasing pressure, the complex structure of ambient liquid water appears to approach that of a simple liquid [6]. An experiment by Yoshida et al. [11] on one particular methanol–water mixture, with 30 mol% of alcohol in the supercritical state at 618 K and 0.1 GPa, may be mentioned as a vague preliminary; note, however, that the thermodynamical state described in [11] does not have much to do with “normal” liquids.

For multi-component systems, where the number of independent total scattering functions should be equal to the number of partial radial distribution functions (PRDFs), obtaining “full” structural information from diffraction measurements is practically impossible. In these cases, computer simulations with interaction potentials will gain more emphasis, and the need for validating results from them will be even more important. Here, we wish to exploit a possible bridge between diffraction experiments and interaction potential models by scrutinizing the performance of well-known classical force fields at elevated pressures. We stress that pressure is an outstanding thermodynamic variable in this respect: increasing pressure directly probes the appropriateness of repulsive interaction potentials.

In what follows, experimental results, in the form of total scattering structure factors as a function of pressure up to 1.2 GPa, are presented. The interpretation of experimental data by molecular dynamics simulations is then described. Then, experimental and computer simulation methods applied in this work are mentioned. Finally, conclusions are drawn. 

## 2. Results and Discussions

Number densities of the mixtures as a function of temperature were determined by the present molecular dynamics simulations, as shown in Table 1, for the calculations using the TIP4P/2005 water model [12]. These density values were systematically used while obtaining structural results described in the remaining parts of this work.

To the best of our knowledge, the only experimental study on the pressure-dependent densities of methanol–water liquid mixtures is that of Kubota et al. [13], which measured molar volumes up to about 0.2 GPa. For the 50 mol% mixture at 0.15 GPa, which is the only point comparable with the present calculations, the computed value was less than 2% higher than the measured one: this is quite a good agreement.

In order to gain more confidence in our simulated density data, we also considered the use of the Tait equation [14]. The authors of [14] state, rather explicitly, that the modified Tait equation is excellent for hydrocarbon liquids up to 150 to 200 MPa, whereas it works much less well for hydrogen-bonded liquids. Here, we had hydrogen-bonded liquid mixtures measured between 150 and 1200 MPa, i.e., the validity of the Tait equation is questionable. Still, simulated values of the pressure-dependent molar volumes follow the predictions of the Tait equation up to 1.2 GPa (not shown) over the entire composition range. 

Finally, it may be in order to mention that our earlier simulated density values [3] agreed very well with measured data over the full composition range at atmospheric pressures. That is, the potential models applied in the present work proved to be accurate enough under standard circumstances (298 K, 1 bar).

### 2.1. Experimental Data: Total Scattering Structure Factors

**Figure 1:**
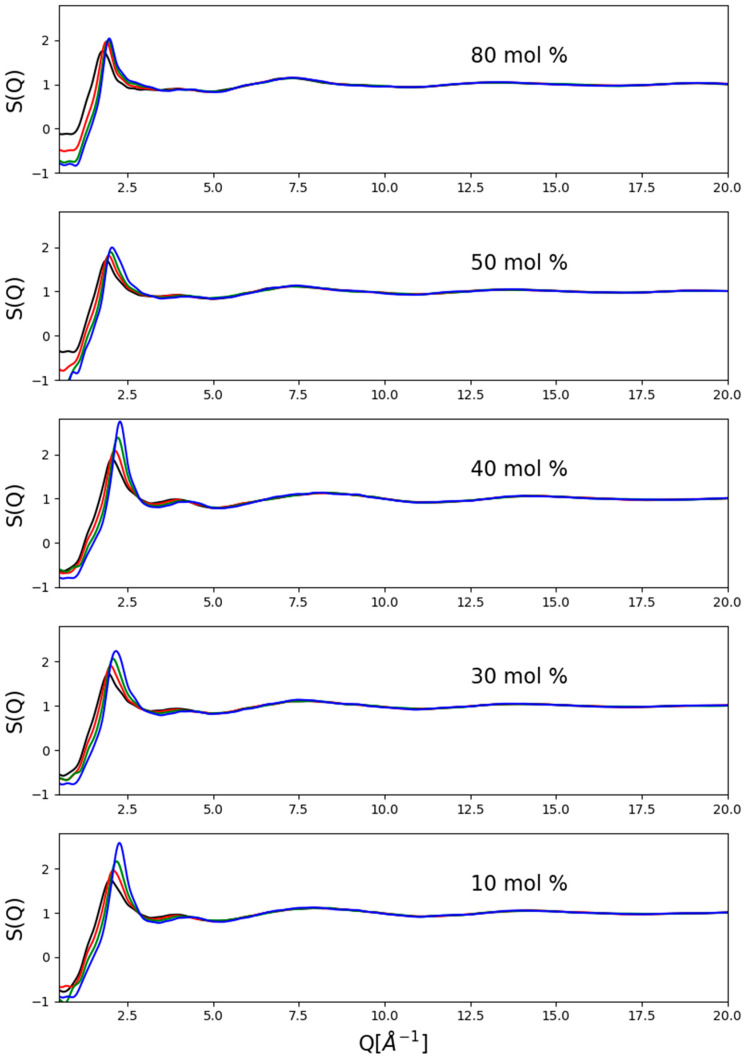
Measured total scattering structure factors of selected methanol–water mixtures at room temperature as a function of pressure between 0.15 and 1.2 GPa. Note that pressure-induced changes were more apparent at lower methanol concentrations (below x_Me_ = ca. 0.5). Black lines: 0.15 GPa; red: 0.4 GPa; green: 0.8 GPa; blue: 1.2 GPa.

A representative set of corrected measured data, in the form of total scattering structure factors up to the scattering variable value of 20 Å^−1^, are presented in Figure 1. Clearly, there were some residual inconsistencies, particularly concerning the region of the lowest Q-values (below ca. 1 Å^−1^), that were due to the immense difficulties with precise corrections at high pressures. These problems clearly indicate the need for further efforts concerning data correction procedures. A couple of general observations may still be made:

(1) There was essentially no pressure dependence above 5 Å^−1^, which may be taken as an indication that the molecular structure was not deformed. This is fully consistent with the observation of Weitkamp et al. [16] concerning the structure of pure methanol at high (up to 0.9 GPa) pressures, who found that the structure factor changes as a response to pressure only at low momentum transfer values.
(2) The most general trend with increasing pressure appeared to be the consistent shift of the position of the first maximum towards higher scattering variable values.
(3) Pressure-induced intensity changes were more visible below a methanol content of about 50 mol%.
(4) Intensities of the first maxima grew with pressure more or less monotonously at a given composition.
(5) High-pressure first-maxima intensities were, in general, higher at low alcohol concentrations, but no clear trend could be spotted.
(6) Since visible changes could only be found below about 6 Å^−1^, in what follows, only the region below this value will be focused on.

### 2.2. Computer Simulations: Comparisons with Measured Data

**Figure 2:**
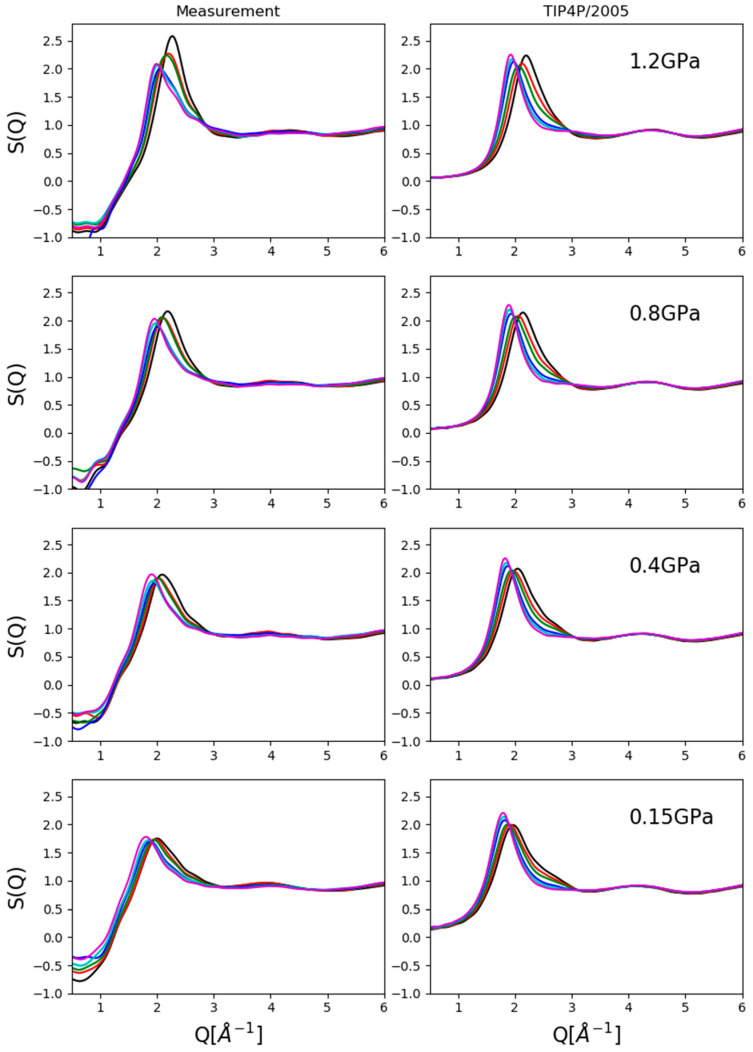
Comparison of measured data and computer simulation results with the TIP4P/2005 water potential as a function of composition at given pressure values. General trends, like the shift of the first peak position and the emerging high Q shoulder on the first maximum, were reproduced satisfactorily, although exact intensities were not. Black: 10 mol% methanol; red: 20 mol%; green: 30 mol%; blue: 50 mol%; cyan: 60 mol%; magenta: 70 mol%.

**Figure 3:**
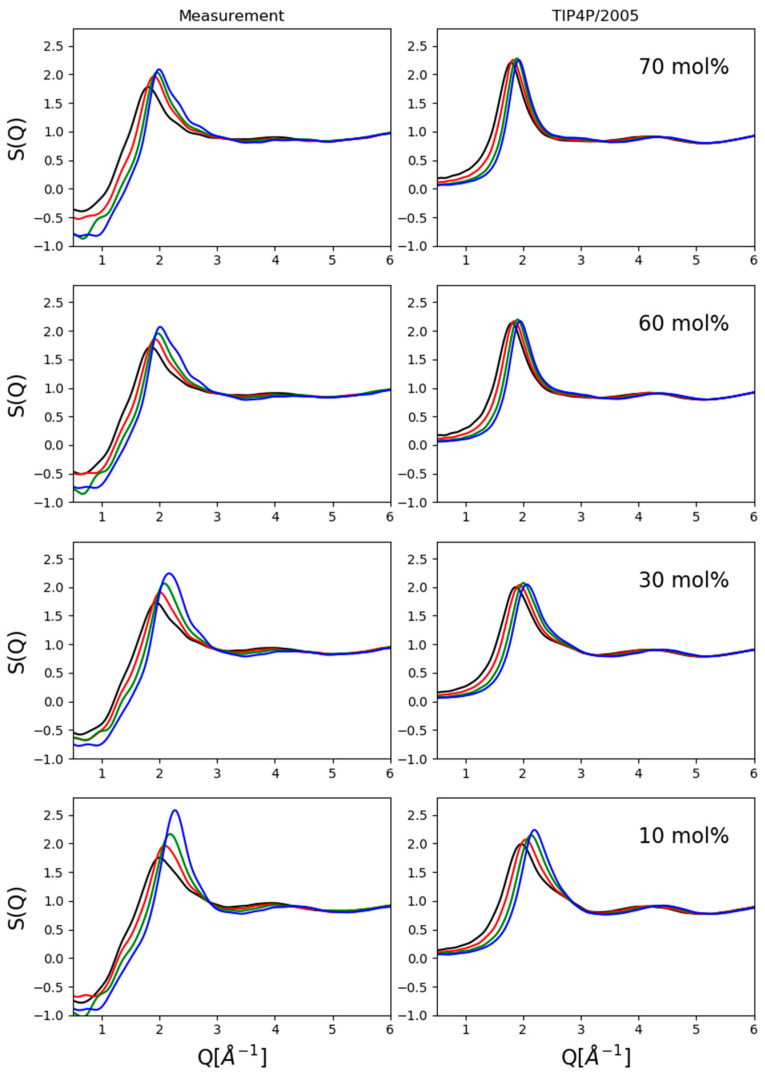
Comparison of measured data and computer simulation results with the TIP4P/2005 water potential as a function of pressure at given compositions. The upward shift of the first peak position with growing pressure was captured satisfactorily. Black lines: 0.15 GPa; red: 0.4 GPa; green: 0.8 GPa; blue: 1.2 GPa.

Two kinds of comparisons are shown here: (1) composition dependence at a given pressure value (Figure 2) and (2) pressure dependence for a given composition (Figure 3). 

Looking at composition dependence (Figure 2), apparent differences can easily be spotted between measured and simulated total structure factors, particularly in terms of exact intensities and even of trends concerning intensities of first maxima. As mentioned previously, these may have been caused by imperfections in the correction process but also, by imperfections of the interatomic potential functions used. Both issues will have to be handled in the future so that the match between experiment and simulation can be called quantitative.

On the other hand, two trends were clearly reproduced: (1) the shift towards lower Q-values of the position of the first maximum with increasing alcohol concentration and (2) the diminishing shoulder on the high-Q side of the first maximum with decreasing alcohol concentration. These observations allowed us to follow and analyze particle configurations in some detail so that valid conclusions could be drawn concerning the pressure dependence of the structure of methanol–water liquid mixtures.

Figure 3 compares experimentally obtained and simulated total scattering structure factors at four compositions as a function of pressure. Again, the upward shift (in terms of Q) of the first peak position with increasing pressure was captured as well as the presence of a shoulder on the high-Q side of the same maximum at lower alcohol concentrations. Agreement with experimental data in terms of intensities was unsatisfactory, and therefore, conclusions were drawn only concerning structure-related tendencies (if any could be identified) with increasing pressure.

### 2.3. Analyses of Simulated Structures: The Effect of Pressure on the Structure

**Figure 4:**
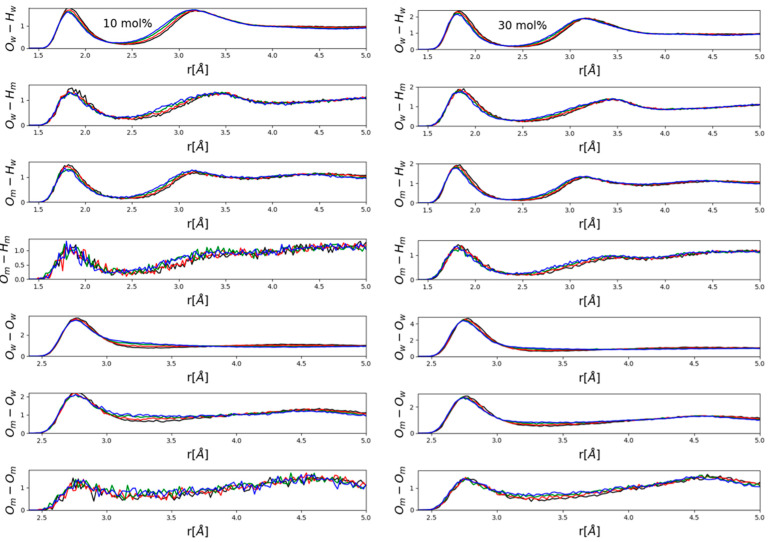
Hydrogen-bonding related partial radial distribution functions in mixtures with low alcohol contents, 10 (left panel) and 30 (right panel) mol% methanol. Black lines: 0.15 GPa; red: 0.4 GPa; green: 0.8 GPa; blue: 1.2 GPa. Indices “m” and “w” refer to “methanol” and “water”, respectively.

**Figure 5:**
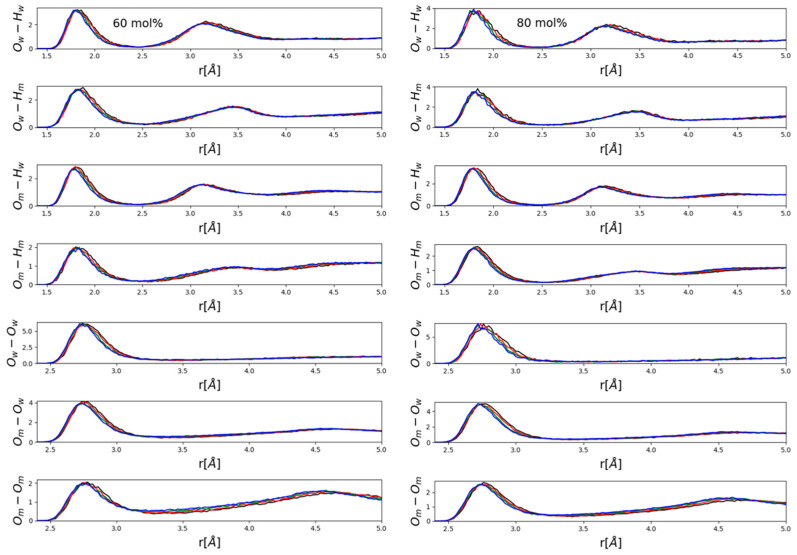
Hydrogen-bonding related partial radial distribution functions in mixtures with high alcohol contents, 60 (left panel) and 80 (right panel) mol% methanol. Black lines: 0.15 GPa; red: 0.4 GPa; green: 0.8 GPa; blue: 1.2 GPa. Indices “m” and “w” refer to “methanol” and “water”, respectively.

Figure 4 and Figure 5 present partial radial distribution functions (PRDFs) that are related to hydrogen bonding. In general, no apparent large effects emerged, although some systematism in terms of the observable small, gradual changes (peak shifts in the order of a few tenths of an Å) was obviously present. Perhaps somewhat surprisingly, intensities did not appear to be affected.

It seems to be instructive to contrast PRDFs of water- (Figure 4) and methanol- (Figure 5) rich mixtures. The main difference is that in water-rich mixtures (Figure 4) the main effect was a visible shift of the second maxima of various OH PRDFs towards lower distances with increasing pressure, while this modification disappeared almost entirely in methanol-rich mixtures (Figure 5). Instead, it was the position of the first maximum of each PRDF shown, OH and OO PRDFs alike, that shifted very slightly towards lower distances with increasing pressure. A possible explanation will be put forward after looking at ring-size distributions (see below). We wish to note, however, that the shortening of intermolecular OH and OO distances that are related to hydrogen-bonding was consistent with the observation of Vondracek et al. [17], made by terahertz spectroscopy on pressurized pure water, that hydrogen bonds may just be shortened as a result of high pressure (i.e., H-bonds are not necessarily broken under pressures of even 1 GPa). 

**Figure 6:**
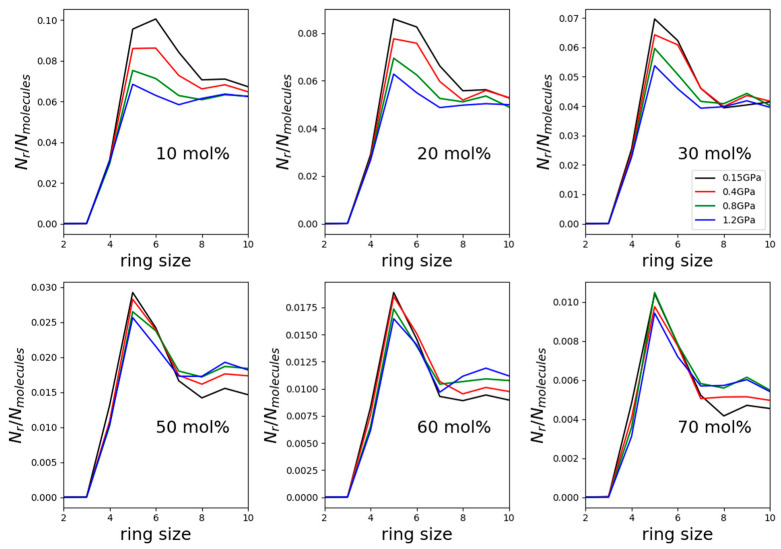
Primitive hydrogen-bonded rings, as represented by the ratio of molecules that participate in small ring-like structures, in computer models of water–methanol mixtures as a function of pressure. Black lines: 0.15 GPa; red: 0.4 GPa; green: 0.8 GPa; blue: 1.2 GPa. Note that the number of cyclic structures fell by one order of magnitude as methanol concentration grew from 10 to 70 mol%.

Distributions of primitive hydrogen-bonded cyclic entities (rings) were determined in each considered mixture for cycles containing at most 10 molecules. Selected results, using the TIP4P/2005 water model, are shown in Figure 6.

The most frequent kinds of rings were the five-membered ones, with the exception of the mixture with the least methanol (10 mol%) at the lowest pressure. In this latter solution, and at the already elevated pressure of 0.15 GPa, six-membered rings were the most common, just like in water-rich methanol–water mixtures under atmospheric pressure and at temperatures between room temperature and freezing point [5]. That is, the relatively low pressure value of 150 MPa seems to be sufficient for modifying the preferred hydrogen-bonded cyclic assembly. In other words, hydrogen bonding, as expected, is influenced by pressure quite considerably.

At the lowest pressure value, the number of hydrogen-bonded rings decreased by roughly one order of magnitude as methanol concentration increased from 10 to 70 mol%, as already recently reported for atmospheric pressure by Pethes et al. [6]. What is more eye-catching is that in the mixture with 10 mol% methanol, the number of cycles decreased by nearly 40% during the pressure increase from 0.15 to 1.2 GPa, while on the alcohol-rich side of the composition range, the decrease was only by about 10%. Both observations can be associated with water molecules that are capable of forming rings more easily than methanol molecules (see also [5,6]). 

Now, we return to the above findings concerning partial radial distribution functions (Figure 4 and Figure 5). The more water molecules there are in a mixture, the more hydrogen-bonded cycles may be formed; when pressure is exerted, the easiest way a hydrogen-bonded system may respond is to break these larger objects that are held together by a secondary intermolecular force. This way, most of the individual hydrogen bonds do not need to change. As a result of breaking the rings, second (and third, etc.) neighbors approach each other more closely—and this is why in water-rich mixtures, it is the second neighbor’s O-H distances that decrease somewhat under high pressures (see Figure 4). In methanol-rich mixtures, on the other hand, there are not enough hydrogen-bonded cyclic structures for this “mild” response to high pressure to be effective; as a result, it is the individual hydrogen bonds that must shrink (cf. also [17]). That is, it must be the first neighbor’s hydrogen-bonding (O-H and O-O alike) distances that shorten under high pressure—exactly as displayed in Figure 5. 

It is perhaps interesting to compare the above composition-dependent duality with earlier conjectures based on NMR results of pure methanol at high pressures [18]: it was found that the extent of hydrogen bonding decreases with increasing pressure, whereas the microscopic picture obtained via first-principles simulations suggested otherwise. We believe that the phenomenological interpretation of NMR experimental results [18] may not have been sufficient for revealing the complex scenario suggested here.

The above discussion is consistent with high-pressure diffraction measurements on liquid water by Soper et al. [9] (cf., in particular, Figure 2 of that reference) and Katayama et al. [10] (cf. Figure 3 of that publication).

## 3. Methods

### 3.1. Neutron Diffraction Experiments

Mixtures of deuterated methanol (isotopic purity better than 99.5%, by Sigma-Aldrich, Budapest, Hungary) and heavy water were prepared with methanol molar ratios, *x_Me_*, of 0.0, 0.1, 0.2, 0.3, 0.4, 0.5, 0.6, 0.7, 0.8 and 1.0.

Neutron diffraction experiments were conducted using the PLANET instrument [19] (beamline: BL11) of the J-PARC Spallation Neutron Facility (Tokai-mura, Japan) using a six-axis press [20]. Samples were encapsulated in Al cubes with a cylindrical sample space of 6 mm × 6 mm. Data were collected at 0.15 GPa, 0.4 GPa, 0.8 GPa and 1.2 GPa at room temperature for each sample as well as for the empty container and vanadium standard (in the same Al container). Generated pressure was estimated from the load applied to the cell, based on the load–pressure curve determined beforehand. The uncertainty of the pressure was considered to be less than 10%. The description of the sample holder, along with the experimental setup, appears in a recent publication [21].

Raw data were handled by a protocol developed by the instrument scientists of the PLANET instrument (see also in [21]).

### 3.2. Molecular Dynamics Computer Simulations

A series of molecular dynamics simulations were conducted in the (NpT) ensemble, using the GROMACS software package [22], code version 2018.2 [23]. Flexible methanol molecules were represented by the OPLS-AA force field [24] and were mixed with rigid (1) TIP4P/2005 [12] or (2) SPC/E [15] water molecules. First, randomly oriented water then methanol molecules were placed in the simulation box, using the “insert molecule” and the “solvate” routines of GROMACS, respectively. Proper mixing of the constituents was achieved by the equilibration scheme applied (see below). The time step was 0.2 fs. To deal with long-range electrostatics, the particle-mesh Ewald algorithm [25,26] was used with a 20 Å cut-off. The same cut-off was set for van der Waals interactions. During the simulations, the following sequence was carried out for each concentration and pressure (p):

(i). 5000 molecules in total, with the appropriate composition, were placed in the simulation box;
(ii). energy minimization;
(iii). NVT equilibration at 400 K for 200 ps at atmospheric pressure;
(iv). NpT equilibration at 400 K and 0.2 GPa for 200 ps;
(v). NpT equilibration at 400 K and given p for 200 ps;
(vi). NpT equilibration at 300 K and given p for 200 ps;
(vii). NpT equilibration at 300 K and given p for 1 ns;
(viii). NpT production, run at 300 K and given p for 2 ns, saving a configuration at each 100 ps.

For steps (iii)–(vi), the Berendsen thermostat [27] with a coupling time of 2.0 ps was used; for steps (iv)–(vi), the Berendsen barostat with a coupling time of 2.0 ps was used; whereas for steps (vii) and (viii), the Nose–Hoover thermostat [28,29] and Parrinello–Rahman barostat [30] were used, with coupling times of 2.0 ps for both. Some calculations were repeated with a barostat coupling constant that was 10 times larger than the thermostat coupling constant: in terms of the quantities determined in this work, no differences could be observed at all. The above scheme, that may appear far too complicated, contributed to proper mixing of the constituents and to the convergency of the system at the required level.

Having no better means for learning the densities of mixtures at the high pressures applied during this study, it was the NpT MD calculations that provided these data for evaluating experimental diffraction data as well.

### 3.3. Analysis of the MD Configurations

Partial radial distribution functions were calculated by the computer program “gmx rdf”, which is part of the GROMACS 2018.2 distribution. The total scattering structure factors were obtained by calculating the partial structure factors from radial distribution functions (via the usual Fourier transformation) and summarizing them by taking into account the coherent neutron scattering lengths and concentrations of the species using an in-house custom written software.

For the hydrogen bond analyses, the geometric definition of hydrogen bonds was selected (r_OO_ < 3.6 Å; r_OH_ < 2.5 Å; O–H..O angle > 150°), and connectivity lists were extracted from saved trajectories. Then, primitive rings (i.e., cyclic entities) were collected using a custom written software in Python, implementing the algorithm of Yuan and Cormack [31].

## 4. Summary and Conclusions

Neutron diffraction experiments were conducted on (deuterated) methanol and (heavy) water liquid mixtures over the entire composition range and at pressures 0.15, 0.4, 0.8 and 1.2 GPa. To the best of our knowledge, this was the first time that the effects of GPa-scale pressure on the structure of any alcohol–water solution were investigated.

For interpreting measured data, molecular dynamics computer simulations were performed for each composition and at every pressure, using OPLS-AA all-atom potentials for methanol [24] and SPC/E [15] and TIP4P/2005 [12] water force fields. 

Based upon the above experimental and simulation work, the following statements can be made:

(1) The influence of pressure on the measured total scattering structure factors could only be detected below about 5 Å^−1^, in the form of tendentious upward shifts (in terms of the scattering variable, Q) of the position and intensity of the first maximum as a function of increasing pressure. This finding is in line with diffraction results of Weitkamp [16] on pure methanol.
(2) The present molecular dynamics simulations were able to reproduce the shifts of the maxima positions. Intensities proved to be much tougher to capture, which indicates that a better description of short-range interactions is needed for a quantitative interpretation of high-pressure data in water–alcohol mixtures.
(3) Scrutinizing O-H and O-O partial radial distribution functions related to hydrogen bonding, it was established that in mixtures in the water-rich composition range, it was the second maximum of the O-H PRDFs that showed the influence of growing pressure. In methanol-rich compositions, the effect was smaller, albeit visible, on both O-H and O-O PRDFs and apparent on the first maxima.
(4) Size distributions of hydrogen-bonded cyclic entities also exhibited a split behavior depending on the composition: at lower methanol concentrations, the decrease in terms of the number of rings was dramatic, whereas at high alcohol contents, the decrease was almost negligible. This duality can explain the different observations concerning partial radial distribution functions: as long as there are many hydrogen-bonded rings, it is sufficient to break larger structures in response to high pressures.

As a final thought, it has to be admitted that both experimental and computer simulation protocols need to be improved for a quantitative description of the structural changes due to pressure in alcohol–water mixtures. Given the exciting qualitative results provided by the present study, such improvements would certainly be worth the efforts.

Concerning the computer simulation part, the use of polarizable potential functions, both for methanol and water, may appear as a logical, although highly non-trivial, step forward. Later, particularly if polarizable potentials cannot provide satisfactory agreement with experiments, even first-principle molecular dynamics may be attempted; this, however, would require quite a few more orders of magnitude of computational time for the set of experimental data presented here.

## Figures and Tables

**Table 1:** Atomic number densities, in units of Å^−3^, of methanol–water mixtures as a function of pressures at room temperature, as obtained from NpT molecular dynamics simulations with the TIP4P/2005 [12] water potential (MeOH: methanol). (Note that similar MD simulations have been conducted with the SPC/E [15] water model, as well, from which nearly identical density values were obtained.).

p[GPa]	Pure Water	10 mol% MeOH	20 mol% MeOH	30 mol% MeOH	40 mol% MeOH	50 mol% MeOH	60 mol% MeOH	70 mol% MeOH	80 mol% MeOH	90 mol% MeOH	Pure MeOH
0.15	0.1074	0.1044	0.1034	0.1025	0.1019	0.1009	0.1001	0.0994	0.0985	0.0976	0.0967
0.4	0.1129	0.1114	0.1101	0.1092	0.1085	0.1078	0.1071	0.1065	0.1058	0.1053	0.1047
0.8	0.1214	0.1194	0.1179	0.1167	0.1159	0.1152	0.1146	0.1139	0.1135	0.1130	0.1125
1.2	0.1365	0.1252	0.1236	0.1224	0.1214	0.1206	0.200	0.1194	0.1189	0.1179	0.1179

## Data Availability

The data presented in this study are contained within the article. The data are also available from the corresponding author in numerical format upon reasonable request.

## Sample Availability

Samples of the deuterated compounds have been contaminated by hydrogen over the past two years, so they are not available. Deuterated methanol and water are both commercially available from the supplier specified in the text.
